# The OncoLifeS data-biobank for oncology: a comprehensive repository of clinical data, biological samples, and the patient’s perspective

**DOI:** 10.1186/s12967-019-2122-x

**Published:** 2019-11-14

**Authors:** Grigory Sidorenkov, Janny Nagel, Coby Meijer, Jacko J. Duker, Harry J. M. Groen, Gyorgy B. Halmos, Maaike H. M. Oonk, Rene J. Oostergo, Bert van der Vegt, Max J. H. Witjes, Marcel Nijland, Klaas Havenga, John H. Maduro, Jourik A. Gietema, Gertruida H. de Bock

**Affiliations:** 1 Department of Epidemiology, University Medical Center Groningen, University of Groningen, Groningen, The Netherlands; 2 Department of Medical Oncology, University Medical Center Groningen, University of Groningen, Groningen, The Netherlands; 3 Department of Pathology, University Medical Center Groningen, University of Groningen, Groningen, The Netherlands; 4 Department of Pulmonary Diseases, University Medical Center Groningen, University of Groningen, Groningen, The Netherlands; 5 Department of Otorhinolaryngology, Head and Neck Surgery, University Medical Center Groningen, University of Groningen, Groningen, The Netherlands; 6 Department of Obstetrics and Gynaecology, University Medical Center Groningen, University of Groningen, Groningen, The Netherlands; 7 Department of Oral and Maxillofacial Surgery, University Medical Center Groningen, University of Groningen, Groningen, The Netherlands; 8 Department of Haematology, University Medical Center Groningen, University of Groningen, Groningen, The Netherlands; 9 Department of Surgery, University Medical Center Groningen, University of Groningen, Groningen, The Netherlands; 10 Department of Radiation Oncology, University Medical Center Groningen, University of Groningen, Groningen, The Netherlands

**Keywords:** Neoplasms, Database, Biobank, Quality of life, Genetics, Proteins, Imaging

## Abstract

**Background:**

Understanding cancer heterogeneity, its temporal evolution over time, and the outcomes of guided treatment depend on accurate data collection in a context of routine clinical care. We have developed a hospital-based data-biobank for oncology, entitled OncoLifeS (*Oncological Life Study: Living well as a cancer survivor*), that links routine clinical data with preserved biological specimens and quality of life assessments. The aim of this study is to describe the organization and development of a data-biobank for cancer research.

**Results:**

We have enrolled 3704 patients aged ≥ 18 years diagnosed with cancer, of which 45 with hereditary breast-ovarian cancer (70% participation rate) as of October 24th, 2019. The average age is 63.6 ± 14.2 years and 1892 (51.1%) are female. The following data are collected: clinical and treatment details, comorbidities, lifestyle, radiological and pathological findings, and long-term outcomes. We also collect and store various biomaterials of patients as well as information from quality of life assessments.

**Conclusion:**

Embedding a data-biobank in clinical care can ensure the collection of high-quality data. Moreover, the inclusion of longitudinal quality of life data allows us to incorporate patients’ perspectives and inclusion of imaging data provides an opportunity for analyzing raw imaging data using artificial intelligence (AI) methods, thus adding new dimensions to the collected data.

## Background

Cancer is mainly considered an age-related disease [1], with more than half of all cancers diagnosed in people older than 70 years [2]. As life expectancy has increased, so too have the incidence and mortality of cancer, resulting in cancer becoming a leading cause of death in many European countries [3]. Indeed, cancer has now overtaken cardiovascular disease as the main cause of death in twelve of these countries [3].

Cancer is a complex disease with more than 1 million known genotypes [4]. Patients with cancer often differ genotypically and phenotypically [5], resulting in marked variabilities in the required management and treatment response. Therefore, personalized treatment approaches are increasingly being used for specific cancers. Although randomized clinical trials (RCTs) are generally considered the best approach for evaluating such treatment approaches, primarily because they reduce the risks of bias, the strict inclusion criteria also limit their generalizability [6, 7]. An alternative source of relevant information is observational research [8, 9].

Observational study involves collecting routine clinical data about carefully selected patient groups, and this can be combined with genetics and omics data from biological specimens [10]. Indeed, understanding cancer heterogeneity, its temporal evolution over time, and the outcomes of guided treatment depend on accurate data about patient characteristics and their clinical management. Linking these routine clinical data to preserved biological specimens can then enable reproducible research capable of discovering biomarkers of cancer and/or treatment response, evaluating personalized treatment approaches, and increasing patient awareness [11]. Including quality of life assessments may provide relevant information on how cancer and its treatment affects the subjective well-being of patients.

We have established a hospital-based data-biobank, entitled OncoLifeS (*Oncological Life Study: Living well as a cancer survivor*) to link routinely collected clinical data with preserved biological specimens and quality of life assessments. The OncoLifeS data-biobank has been designed to facilitate cancer research by providing clear phenotypic and genotypic data in a clinical context.

## Results

### Current state of the OncoLifeS data-biobank

As of October 24th, 2019, we had included 3704 patients with cancer in the OncoLifeS data-biobank (Table 1). The average age at inclusion was 63.6 ± 14.2 years and 1892 are females (51.1%). To date, most patients (82.6%) have been included by the three largest tumor working groups, with gynecological oncology contributing 24.6%, lung oncology contributing 22.4%, and head and neck oncology contributing 35.6%. We have also included 45 patients diagnosed with hereditary breast-ovarian cancer (average age, 36.5 ± 11.3 years Four papers using the data of OncoLifeS has been published in 2019 in peer-reviewed journals so far [12–15].

**Table 1:** Participation in the OncoLifeS data-biobank by tumor working group

Tumor working group	Number of patients	Participation rate 2017–2018
Gynecological oncology	910	94%
Lung oncology	831	64%
Testicular oncology	98	100%
Head and neck oncology	1319	64%
Medical oncology/immunotherapy	77	^a^
Hematological oncology	343	^a^
Neuro-endocrine oncology	45	^a^
Bone and soft-tissue oncology	36	^a^
Hereditary breast-ovarian cancer	45	^a^
Total	3704	

Number of patients included and participation rate for a 2 years period (2017–2018)

^a^Not available, as these groups did include since 2019

We anticipate that approximately 1500 patients will be included in the data-biobank each year from 2019, with the total size therefore expected to reach 10,000 participants by 2023. Overall, about 70% of approached patients have agreed to participate, with the estimated participation rate per tumor working group between 2017 and 2018 shown in Table 1. The other 30% of patients were either not asked or refused to participate, and although the exact numbers are unknown, most of these were not asked. To date, 34 patients (1%) have withdrawn their informed consent. Patient-reported data, including data about quality of life, are collected for about 70–80% of patients depending on a tumor group. The examples of quality of life [12, 14] and imaging [15] data analysis could be found in previous studies.

### Composition of the three largest groups

Of the three largest groups, patients from the head and neck oncology group are the oldest, having a mean age of 68.9 ± 11.9 years at baseline. In this group, more than 50% of the included patients have stage III or IV cancer, with 50% undergoing surgery, 25% undergoing radiotherapy, and 12% undergoing chemotherapy. Biomaterials are available for more than 40% of these patients. The imaging data are available for all patients in this group, except those with very small tumors (T1a) and very small skin cancers.

In the lung oncology group, the average age was 64.5 ± 9.9 years at baseline, with most (90%) having stage III or IV non-small cell lung cancer. More than 70% of the patients in this group received chemotherapy and 40% received radiotherapy; however, only 25% underwent surgery. Biomaterials are available for more than 80% of these patients. The imaging data are available for 95% of patients in this group.

In the gynecological oncology group, the average age was 62.5 ± 14 years at baseline, half of all patients have ovarian carcinoma, and 40% have stage III or IV disease. The rates of surgery, radiotherapy, and chemotherapy in this group are 77%, 20%, and 17%, respectively. Biomaterials are available for 80% of these patients. The imaging data are available for 95% of patients in this group.

## Discussion

The OncoLifeS data-biobank was established in 2014 to provide an infrastructure for clinical cancer research, to facilitate translational research toward more personalized cancer care, and to monitor oncological quality of care outcomes. At the time of writing, we have already established a sizable data-biobank, and this is continuing to grow. The process of building a data-biobank is rarely described and even less often published in peer-reviewed journals, where it can be critically evaluated by external reviewers. We have demonstrated the feasibility of data-biobank building with a relatively low budget by collecting data in routine clinical care. The infrastructure required for the data-biobank has been described, including the procedures needed for data collection, handling, storage, and access. The data from OncoLifeS are managed and available for research purposes according to findable, accessible, interoperable, and re-usable principles [16].

The extensive data in the OncoLifeS data-biobank provides many opportunities for researchers to study cancer at molecular and clinical levels. This includes disease etiology, disease processes, response to cancer treatment (including longitudinal quality of life data), and the short- and long-term side-effects of treatment. Five of the main benefits and potential uses of the OncoLifeS initiative are worthy of note. First, the data-biobank covers uncommon cancers and does not focus solely on major cancer types. Second, observational studies based on OncoLifeS data can be used to evaluate the impact of clinical interventions on quality of life. The quality of life data collected over multiple time points in the follow up provides relevant information on subjective patients well-being in the course of treatment and cancer progression. Third, the data can be used to identify molecular and imaging biomarkers that can predict a range of outcomes, such as disease progression and response to various treatments. Availability of raw imaging data linked to other clinical data and biological specimens is a unique features of the OncoLifeS, allowing application of Artificial intelligence (AI) methods for diagnostic and cancer progression research. Fourth, this data-biobank can facilitate translational research and investigations into the course of cancer after a given treatment, thereby helping to identify risk or protective factors, and helping with post-marketing surveillance. Fifth, we can infer trends in health care and related costs from the data.

The OncoLifeS data-biobank is embedded in a large academic hospital, which ensures structured data storage and management, with continued adherence to high legal and ethical standards. Other studies falling under the scope of the “Dutch Medical Research Involving Human Subject Act (WMO 1998)” can adopt the methods of the OncoLifeS initiative for data collection, data storage, and biomaterial collection and processing, provided they have appropriate governance procedures and ethical approvals in place.

We included a population of consecutive people diagnosed either with cancer or with a genetically increased risk of cancer. To improve the relevance to future research, we collected important contextual data with the biological samples, such as patient-reported history, quality of life, and outcomes. The OncoLifeS initiative also involves health care providers in data collection, thereby maintaining continued data access and engagement in research. This facilitates interpretation of research in a clinical context and could optimize care regimens [17]. In contrast to RCT designs, which include highly selected patient populations, our design can provide more generalizable and clinically relevant conclusions [8].

Advances in omics methodologies and big-data analytics in cancer research have led to the emergence of several cancer data-biobanks in Europe and worldwide [18, 19]. Most of these collect DNA, blood, and tissue samples, and/or are specific to a particular cancer or problem (e.g., biomarker discovery). However, few have been linked to clinical data including medical imaging data. In the Netherlands, relevant biobanks are the BOSOM, Maastricht UMC, ORIGO Leiden, the vUMC, and Parelsnoer biobanks, with focuses on breast cancer, head and neck cancer, and colorectal and gastric cancers. Other biobanks include data and tissue collections from big clinical trials (e.g., the TUMOROID trial and the TripleB study) [20]. The Radboud Biobank from Nijmegen is perhaps most comparable to the OncoLifeS data-biobank, providing access to both biological specimens and linked clinical data [21].

Unlike its predecessors, a notable strength of the OncoLifeS data-biobank is that it includes longitudinal quality of life data that will allow patient perspectives to be incorporated in research. Moreover, the OncoLifeS data provide an opportunity to analyze raw imaging data (e.g., computed tomography images) in the rapidly developing field of quantitative imaging (radiomics). Other strengths include the systematic and routine collection of clinical and socioeconomic data that are linked to collected biological specimens. These specimens are often collected and stored without a structured protocol and often vary in quality due to the myriad of factors that influence the collection, processing, and storage of specimens [22, 23]. Our strict protocols for sampling, handling, and storage moderate these factors. Moreover, routine clinical and/or radiological data are often not available or are not linked to specimens in data-biobanks. Therefore, data-biobanks that have standardized and established procedures, such as OncoLifeS, can be linked to other trusted data, thereby improving research collaboration and the quality of cancer research.

The continued functioning of any data-biobank requires funding to cover infrastructure and personnel costs. Although the OncoLifeS data-biobank is currently supported by the University Medical Center Groningen (UMCG), there is no guarantee that this funding will continue in the future. To cover its operational costs, the OncoLifeS must therefore attract the interest and collaboration of other researchers, institutions, and businesses. Collaboration is being promoted through the Groningen Data Catalogue [24], Biomarker Bay [25], and by the Biobanks and Biomolecular Resources Research Infrastructure in the Netherlands [20]. Thankfully, a straightforward administrative process makes the OncoLifeS data-biobank easy to access, which will facilitate collaboration.

Our data-biobanking model ensures that high-quality clinical data linked to biomaterial are available for translational research. The data-biobank respects the requirements of the European General Data Protection Regulation with methods of data collection. and anonymization, together with the consent requirements, allowing data use for analysis. Data from the OncoLifeS initiative can be used to evaluate and improve treatment for patients who may otherwise never be included in clinical trials, providing additional information across a broader spectrum of conditions when compared with other data-biobanks. We plan to expand the data collection to include not only unstructured (free text) data from hospital records but also to add more details about comorbidities. Finally, we also plan to extend the patient-reported outcomes to include measures of pain, fatigue, sleep, depression and anxiety, and the ability to participate in social roles and activities [26].

## Methods

### Aims

The primary aims of the current work were to describe the organization and process of starting our data-biobank of clinical data, biological samples, and quality of life assessments in a clinical setting. We also present some preliminary results to demonstrate the feasibility of this data-biobank. Overall, however, we seek to provide guidance on how to set up and run a successful data-biobank by describing our experiences.

### Setting

The OncoLifeS data-biobank has been embedded within the structure of the UMCG, an academic, medical, tertiary referral center in the north of the Netherlands, covering an area with 3.4 million inhabitants. Oncological care within the UMCG is provided by multidisciplinary tumor working groups that include the specialists needed to provide optimally personalized cancer care. The UMCG has 16 different tumor working groups that manage both low- and high-volume tumors. Treatment decisions are supported by a weekly molecular tumor board focusing on DNA, RNA, and protein aberrations, using novel technology to predict the added value of targeted therapy. The OncoLifeS initiative was established by the Cancer Research Center of the Comprehensive Cancer Center and the Department of Epidemiology in close cooperation with the 16 tumor working groups.

The UMCG is an internationally and nationally recognized expert center for several rare tumors, including head and neck, neurological and neuroendocrine, soft-tissue and bone, gynecological, esophageal and gastric, testicular germ-cell, and some hereditary cancers, as well as mastocytosis and mucosa-associated lymphoid tissue (MALT) lymphoma related to Sjögren syndrome. The UMCG is a partner of the European reference network on rare cancers (EURACAN), and is active in four domains: sarcoma, neuroendocrine tumors, rare gynecological tumors, and testicular germ-cell tumors [27]. At the UMCG, we also offer specialist care to patients with lung cancer who require targeted treatment and we serve as a reference center for cases of acute myeloid leukemia requiring intensive treatment.

### Design

Inclusion in the OncoLifeS data-biobank is prospective and on an ongoing basis. All adult patients (age > 18 years) diagnosed with cancer or with a genetically increased risk of cancer are included, without further exclusion criteria. Informed consent is obtained from patients before inclusion, and the data collection processes are embedded in routine care. All relevant processes are described with standard operating procedures to ensure that the data-biobank is of high quality. Established in 2014, the data-biobank first began with the inclusion of patients diagnosed with head and neck cancer (October 2014), but we soon included patients diagnosed with lung cancer (October 2015) and gynecological cancer (January 2016). After a 3-year consolidation period, other tumor working groups have started to participate, including those for testicular, hematological, brain, and neuroendocrine cancers, and those covering specific patients (e.g., immunotherapy-treated, adolescent, and young adult groups).

### Data protection and regulation

Currently, no specific law in the Netherlands governs data-biobanking, and the OncoLifeS initiative does not fall under the scope of the Dutch Medical Research Involving Human Subject Act (WMO), 1998. We therefore followed the national guideline “Human Tissue and Medical Research: code of conduct for responsible use (2011)” [28], the internal UMCG guidance for data-biobanking (not published; available internally in Dutch only), and the requirements of the European General Data Protection Regulation for scientific research. The data-biobank is being coordinated by a manager (GHdB), coordinator (JN), steering committee (see author list), and independent scientific board (see acknowledgment). Representatives of each participating tumor working group are included in both the steering committee and the scientific board.

The OncoLifeS initiative has been approved by the medical ethics committee of the UMCG (no. 2010/109) and has been ISO certified (9001:2008 Healthcare). It was registered in the Dutch Trial Register under the number: NL7839.

### Informed consent

When patients are invited for a clinical visit, they receive an information leaflet about the OncoLifeS initiative. At their first visit, a physician, nurse practitioner, or a (research) nurse further informs the patient about the OncoLifeS data-biobank and asks if they are willing to participate. If the patient agrees, his or her written consent is obtained for each of the following: (1) to use all clinical, patient, tumor, treatment, and outcome data; (2) to collect biomaterials; (3) to collect clinically relevant patient-reported data (e.g., data on lifestyle and quality of life); (4) to store data and biomaterial infinitely; (5) to obtain data from other sources (e.g., general practitioners, pharmacists, and other hospitals); (6) to link with other data(bio)bases (e.g., municipal registration, Central Bureau for Statistics, the Netherlands Comprehensive Cancer Center, the nationwide registry of histo- and cytopathology in the Netherlands, and LifeLines (a large cohort study of a random sample of 10% of inhabitants in the north of the Netherlands) [29]; (7) to allow research to improve outcome of cancer treatment and living as a cancer survivor; (8) to use residual tissue, bone marrow, and blood samples; (9) to allow collaboration between medical doctors and national or international organizations/companies; and (10) to publish results in scientific journals. Patients are also asked to give their permission to be contacted during follow-up either by researchers if additional data is needed or by a physician if there are unanticipated clinically relevant findings. Participants are informed that they retain the right to withdraw their consent at any stage.

### Data and biomaterial collection

Clinical data are collected by physicians during routine clinical care. This data includes patient characteristics, comorbidities, oncological diagnosis and staging, diagnostic details (e.g., pathology reports and radiological images), and treatments (see Table 2). Baseline data are retrieved from the hospital’s electronic health care record system (Epic, Epic Systems Corporation, Verona, WI). Data concerning tumor stage (TNM staging is according to the International Classification of Diseases for Oncology) and cancer treatment is confirmed by the different participating multidisciplinary tumor working groups. A comparable approach has successfully been used by the UMCG family cancer clinic for patients at increased risk of breast and ovarian cancer [30].

**Table 2:** Clinical data collected from the electronic medical records of the hospital

General patient information	Identification number Gender Date of birth Date of death Postal Code
Clinical history	Comorbidity, Clinical history of cancer management
Comorbidity	The Adult Comorbidity Evaluation 27 (ACE-27) score
Clinical and pathological diagnosis	Date of diagnosis Tumor type Radiological images Clinical TNM classification Pathological diagnosis Stage Intention to treat (curative/palliative) Recurrences Follow-up status (status dead or alive) and related date
Surgical treatment	Type of surgery Date of surgery
Radiotherapy	Type of radiotherapy Date of start radiotherapy Date of end radiotherapy Number of fractions and Gy per fraction
Systemic therapy	Type of treatment Date of start treatment Date of end treatment Scheme of treatment

Patient-reported data are collected via questionnaire at baseline (see Table 3), including data on family history of cancer, lifestyle, social status, quality of life, and comorbidities. For patients aged 65 years and older, we also include evaluations of daily living activities and frailty. The baseline questionnaires are sent to participants by post or email within one week of gaining informed consent. Data on quality of life are collected by questionnaire at baseline and at 6, 12, 18, and 24 months after the start of treatment.

**Table 3:** Data collected from patient-completed questionnaires

Family history	Questionnaire developed by OncoLifeS team Cancer in first- and second-degree family members, kind of cancer
Lifestyle	Questionnaire developed by OncoLifeS team Smoking habit Alcohol intake Malnutrition Universal Screening Tool (MUST)
Social conditions	Questionnaire developed by OncoLifeS team Country of birth Marital status Born children Living situation Education Working habits
Quality of life	European Organisation for Research and Treatment of Cancer Quality of Life Questionnaire (30 questions) (EORTC QLQ-30) Cancer Quality of Life EORTC Head and Neck module (35 questions) (EORTC QLQ-35)
Daily living activities	Instrumental Activity of Daily Living Questionnaire (IADL-Q)
Frailty	Groningen Frailty Indicator (for patients 65 years and older) G8 Questionnaire (for patients 65 years and older)
Mental state status	Geriatric Depression Scale (GDS-15) questionnaire (only for patients with head and neck cancer) Mini-Mental State Examination (MMSE; only for patients 65 years and older with head and neck cancer)

Several biomaterials are routinely collected during clinical care. The following are collected and stored for future use: serum, heparin-plasma, heparin-plasma (for cell isolation) ethylenediaminetetraacetic acid (EDTA) plasma, + buffy coat (DNA), Genomic DNA (by whole blood collected in EDTA tubes), plasma for cell-free DNA (by whole blood collected in Streck tubes), RNA (by whole blood collected in PAXgene tubes), bone marrow, feces, urine, tumor tissue, and tissue adjacent to a tumor (see Table 4).

**Table 4:** The processing and storage of biomaterials in the OncoLifeS data-biobank

Serum	10 mL serum clot tube (BD 367896)	Centrifuge (10 min, 1300*g*, RT)—aliquot serum 5×, store at − 80 °C
EDTA-plasma + buffy coat (DNA)	10 mL K2-EDTA tube (BD 367525)	Centrifuge (10 min, 1300*g*, RT), aliquot buffy coat 1×, aliquot EDTA-plasma 5× store at − 80 °C
Heparin-plasma	10 mL Li-heparin tube (BD 367526) 2 × 10 mL Li-heparin tube (BD 367526)	Centrifuge (10 min, 1300*g*, RT), aliquot heparin-plasma 5× store at − 80 °C Monoculear cell isolation over lymphoprep, 100e6 MNCs per vial, stored in liquid nitrogen
Plasma (for cell-free DNA)	2 × 8 mL tube (Streck 218997)	Centrifuge (20 min, 1600*g*, RT), collect plasma, centrifuge (10 min, 16,000*g*, RT), aliquot cell-free DNA plasma 10×, store at − 80 °C
Whole blood (for DNA)	10 mL K2-EDTA tube (BD 367525)	No processing, store tube at − 80 °C
Whole blood (for RNA)	Paxgene tube (BD 762165)	No processing, store tube at − 80 °C
Bone marrow	3 × 10 mL syringe with Na-heparin	Isolation of mononuclear cells by density centrifugation, controlled freezing of isolated mononuclear cells, store in liquid nitrogen
Feces swab in glycerol b	Glycerol swab (Copan Custom kit M1016004)	No processing, store tube with swab at − 80 °C
Feces	3 × 2.0 mL cryotubes (Greiner 122280)	No processing, store cryotubes at − 80 °C
Urine	9.5 mL urine tube (BD 365000)	Centrifuge (10 min, 1300*g* RT), aliquot urine 6×, store at − 80 °C
Tumor tissue (for paraffin embedding)	Tissue fixed in formalin	Tissue embedded in paraffin, store at RT
Tissue adjacent to a tumor (for frozen section)	Tissue in tin	Tissue in tin, store at − 80 °C
Tissue adjacent to a tumor (for paraffin embedding)	Tissue fixed in formalin	Tissue embedded in paraffin, store at RT
Healthy tissue (for frozen section)	Tissue in tin	Tissue in tin, store at − 80 °C

*RT* room temperature

During follow-up, we have sought to include long-term outcome data regarding response to cancer treatment, treatment complications (including side-effects), recurrence, new cancers, disease-specific survival, overall survival, and patient-reported quality of life. Patient survival is evaluated monthly by linkage to municipal death registrations. Data from the OncoLifeS data-biobank can also be linked to other sources of data to gain insights into outpatient medicine used (e.g., pharmacy data), and treatment outcomes (e.g., survival in years), which can provide long-term outcome data. The linkage procedure differs for each data source and is performed by a trusted third party that is also responsible for anonymization.

### Data and biomaterial handling

Clinical and patient-reported data are stored in a central database, with data management performed by a UMCG-developed application named Utopia. This application handles all necessary data management processes, including the integration of patient and laboratory data and the logistics for sending out study questionnaires. Utopia was developed using Microsoft C# and all data is stored on a Microsoft SQL Server. Authentication and authorization is via an Advantage Database Server. Full audit-trail support has been built into optimize data quality.

Imaging data of the OncoLifeS participants is exported from imaging devices (e.g. Computed Tomography (CT) or Magnetic Resonance Imaging (MRI) scanner) to the Picture Archiving and Communication System (PACS), which is a medical imaging technology providing economical storage and convenient access to images from multiple modalities. The images are stored in PACS indefinitely. When required by a researcher, the images could be retrieved from PACS and copied to a secured environment, where researchers could access raw images for analysis using various software, such as Syngo.via, TeraRecon, etc.

Biomaterials are collected during clinical care. Blood samples are sent to the laboratory for both routine diagnostics and storage. Urine samples are collected by participants using a collection kit and are sent to the laboratory for storage. Fecal samples are also obtained via a collection kit, but the samples are delivered at the next follow-up visit. Tissue samples are collected in an operation theater during surgery or in an outpatient clinic during biopsy. Bone marrow aspirates are taken during routine procedures. All blood, fecal, and urine samples are labeled and sent to the central laboratory of the UMCG. The biomaterials are processed according to standard operating procedures and are stored accordingly (Table 4). Long-term storage is guaranteed by a centralized freezing service offered by the LifeStore facility of the UMCG. Biomaterials are processed according to standard operating procedures and stored at the Department of Pathology and Department of Hematology. Storage devices (both freezers and liquid nitrogen storage) are 24/7 controlled/monitored by a professional service for quality control XiltriX. Quality control for biomaterials is covered by standard clinical practice and regular quality control is in progress for the biomaterials collected since the OncoLifeS data-biobank was established.

Tumorous tissue samples and tissue adjacent to a tumor are transported to and handled by the Department of Pathology. Tumor tissue undergoes standardized macroscopy to assess its suitability for biobanking, with emphasis placed on the need to ensure that biobanking does not interfere with primary diagnosis. The dedicated OncoLifeS samples are then coded separately from the clinical workflow and either formalin-fixed and paraffin embedded or kept as fresh frozen tissue at − 80 °C. These samples are stored indefinitely until needed for research or diagnostic purposes. In addition, the clinical paraffin blocks are stored for 110 years by the Department of Pathology of the UMCG, and these are available for study purposes provided sufficient tissue remains for future diagnostic assessment. Bone marrow aspirates are transported to and handled by the Department of Haematology. The aspirates for the biobank are used to isolate bone marrow cells, which are then frozen and stored in liquid nitrogen at − 196 °C.

### Data and biomaterial access

Interested stakeholders can submit a research proposal to the coordinator of the OncoLifeS data-biobank and the involved tumor working group. The OncoLifeS scientific advisory board, which comprises representatives of each participating tumor working group, will then review the proposal. This board then advises the OncoLifeS steering committee on whether to approve requests for the use of clinical data and/or biomaterials. If approved, the project coordinator retrieves anonymized data or biomaterials from the OncoLifeS database or biobank. A protected workspace is provided for access to and analysis of data regarding cancer biomarkers, treatment response, and treatment effects for different patient outcomes, including quality of life. This can also be used to give health care providers performance-related feedback and to monitor oncological care quality. If biomaterials are requested, agreements are made on how, where, and by whom they will be analyzed, and results from these analyses will be added to the workspace. On this workstation, the researcher will have access to the set of requested data. In addition, software is provided for data analysis.

### Funding

The OncoLifeS initiative was established with funding from the UMCG and the Cancer Research Centre of the UMCG. The infrastructure for collection, processing, storing, and labeling of biomaterials is partly provided by the UMCG, with the ongoing costs of data collection and biological specimen storage covered by each participating clinical department. External parties will also be asked to cover our expenses and running costs when accessing.

## Data Availability

The OncoLifeS data-biobank follows the requirements of the European General Data Protection Regulation for scientific research. Data from the OncoLifeS database, can be approached in a protected workspace after approval of the steering committee. Interested researchers may contact the OncoLifeS initiative directly (http://www.oncolifes.nl) to inquire about access to the data.

## Abbreviations

RCTs: randomized clinical trials
AI: artificial intelligence
UMCG: University Medical Center Groningen
EURACAN: European reference network on rare cancers
MALT: mucosa-associated lymphoid tissue
EDTA: ethylenediaminetetraacetic acid
PACS: Picture Archiving and Communication System
CT: computed tomography
MRI: magnetic resonance imaging
